# Effect of Oxide Metallurgy on Inclusions in 125 ksi Grade OCTG Steel with Sulfide Stress Corrosion Resistance

**DOI:** 10.3390/ma15134544

**Published:** 2022-06-28

**Authors:** Si Zhang, Yanmei Li, Ping Wang, Fuxian Zhu, Yulong Yang, Bang Xiao

**Affiliations:** 1 Key Laboratory of Electromagnetic Processing of Materials, Northeastern University, Shenyang 110819, China; zsiyang@126.com (S.Z.); yyylll2007@163.com (Y.Y.); chopin1005@163.com (B.X.); 2 State Key Laboratory of Rolling and Automation, Northeastern University, Shenyang 110819, China; zhufuxian@163.com

**Keywords:** sulfide stress cracking (SSC), inclusion, oxide metallurgy, prior austenite grain

## Abstract

The effects of Al deoxidation and Zr deoxidation on the microstructure and properties of sulfide stress corrosion resistant high-strength steel have been investigated. The feasibility of the Zr deoxidation instead of Al deoxidation was confirmed by the thermodynamic analysis of the deoxidation of various elements. The experimental results indicate that the average diameters of the inclusions in Al-Steel and Zr-Steel were 2.45 μm and 1.65 μm, respectively. The Al-Steel and Zr-Steel contained 22.38% and 68.77% inclusions per unit area, respectively, and the fraction of inclusions in the Al-Steel and Zr-Steel with diameters less than 2 μm was about 73.46% and 89.63%, respectively, indicating that the Zr deoxidation process could effectively refine inclusions and promote dispersion. The average diameters of austenite grain for the Al-Steel and Zr-Steel were about 9.1 μm and 8 μm, respectively. The fine particles in Zr-Steel could pin the austenite grain boundaries and clearly refine the grains. The average grain size of tempered martensite was 8.2 μm and 3.8 μm, respectively. The yield strength of the Al-Steel and Zr-Steel was 922 MPa and 939 MPa, respectively; the impact energy was 60 ± 6 J and 132 ± 6 J, respectively. Moreover, the fracture time of the NACE-A was from 28 h (Al-Steel) to 720 h (Zr-Steel) without fracture. The experimental steel deoxidized by Zr achieved a simultaneous improvement in strength, toughness and sulfide stress corrosion resistance, and the effect of inclusions on the fracture of the sulfide stress corrosion resistant high-strength steel can be explained by the Griffith theory.

## 1. Introduction

With the increasing requirement for and consumption of oil and natural gas resources worldwide, the exploitation of oil and natural gas is advancing towards the corroded wells which are difficult to exploit. Corrosive wells often contain corrosive gases such as H_2_S and CO_2_, and the corrosion of oil and gas wells by H_2_S is particularly serious [1]. The ordinary oil country tubular goods (OCTG) once applied to the oil and gas wells, which contain H_2_S, will trigger sulfide stress corrosion (SSC) cracking, that is, the sudden fracture of the ordinary steel occurs when the load stress is far less than the yield strength in a wet H_2_S environment (where even the H_2_S partial pressure is very small [2]), causing frequent malignant safety accidents. In practice, the OCTG not only can bear the single or combined corrosion of H_2_S, CO_2_ and Cl^−^, but it can also bear the complex external force exerted by complex geological conditions or a creep formation on it [3]. The stress and ambient temperature will also increase with the depth of the OCTG increasing, and it actually bears a strong external stress during its work; therefore, the research and development of OCTG with a high strength and SSC resistance have attracted much attention.

Previously, many researchers were mainly focused on the effects of the chemical composition and heat treatment process on the mechanical properties and microstructure of OCTG steel. For example, Wang et al. [4] studied the effects of Cr and W contents on SSC resistance and Luo et. al [5] discussed the tempering process on the strength and SSC resistance of Cr-Mo steel; however, few researchers have studied the influence of the metallurgical process on the SSC resistance of OCTG steel since Al deoxidation technology is now the most widely used in conventional smelting, which is the core content of the existing deoxidation technology [6]. As a stronger deoxidizer, Al is added into the furnace in the first step of the deoxidation process, reducing the oxygen content to a very low level; however, the Al deoxidation process will inevitably bring deoxidation products of Al_2_O_3_ [7], which become a kind of non-metallic inclusion in steel [6,7]. Inclusions were usually considered as the irreversible hydrogen traps of H atoms, deteriorating the microstructure, inducing component cracking and reducing the properties of steel [8,9]. It is well known that Al_2_O_3_ and MnS inclusions, which are formed during the steelmaking process [10], often act as pitting corrosion nucleation sites and the main fracture sources and starting points of SSC [11].

The difference in the thermal expansion coefficient and mechanical properties between the inclusions and the steel matrix can affect the stress state and crack of the material [12,13]. Meanwhile, the thermal expansion coefficient of Al_2_O_3_ inclusions is lower than that of the steel matrix, which cause a residual stress in the matrix. These stresses may change the local electrochemical activity and mechanical properties of the matrix/inclusion interface, thus affecting the corrosion and stress corrosion crack initiation [12]. On the contrary, inclusions with a higher coefficient of thermal expansion than the steel matrix, such as MnS, contribute to the formation of micro-cracks between the inclusions and the steel matrix [13]. Al deoxidation products, where some are angular, long and hard, cannot deform with the steel matrix during hot rolling due to their differences from the mechanical properties of the steel matrix, resulting in a stress concentration at the steel/inclusion interface [14,15].

Highly localized stress may lead to hydrogen enrichment [12]. For example, stress localization at the inclusion/matrix interface is a preferential site for crack initiation and hydrogen trapping [16]. The hydrogen atoms that are diffused from the steel surface are enriched at the lattice defects caused by inclusions, forming molecular hydrogen [17]. The steel matrix near an inclusion is subjected to plastic strain, which accelerates its hydrogen charging and embrittlement [18]. When the molecular hydrogen pressure at the top of the inclusion rises above the critical value, SSC cracks will appear, leading to the failure of the sample. It is reported that SSC cracks tend to nucleate at the sites around the hard phases in steels, and the local stress around these particles could exceed the yielding stress and increase the risk of crack initiation [19]. Among them, MnS and Al_2_O_3_ are the most destructive and typical harmful inclusions in SSC, which are the most common SSC crack sources. Jin et al. [20] and Xue et al. [21] showed that hydrogen-related stress corrosion cracking can easily nucleate at the sites around aluminum oxide inclusions.

Some studies [16,22] have shown that the inclusion of fine, spheroidized and dispersed inclusions could help to prevent pitting corrosion and reduce the starting point of SSC cracking. A way to improve the SSC resistance is to better control the non-metallic inclusions by reducing the inclusion sizes and eliminating any elongated inclusions [16]. By increasing the volume fraction of inclusions in steels, the irreversible hydrogen is trapped more efficiently, resulting in an increasing hydrogen pressure at this position, thus improving the SSC of the material. Spheroidized inclusions are prone to be in a stable state, because there is a relatively small local lattice deflection around such inclusions [23]. By reducing the hydrogen accumulation and local stress concentration, spheroidized inclusions improve the SSC resistance of the material.

Raphael et al. [15] confirmed that the oxidation of steel and the titanium and the sulfur content in steel are the main obstacles to the modification of non-metallic inclusions while Guo et al. [24] investigated the method of spheroidizing Al_2_O_3_ inclusions. Meanwhile, Van Ende MA et al. [25,26] further refined the peripheral elements surrounding Al_2_O_3_, suggesting that fine and spherical inclusions can improve strength and toughness and compared with non-refined and non-spheroidized inclusions, the hydrogen embrittlement is reduced or not caused. Wael et al. [27] tested different proportions of CeO_2_ and Al_2_O_3_ inclusions, and clarified that the surface of the Al_2_O_3_ was covered with fine CeO_2_ particles, forming a layer to inhibit the interaction between the metal and the Al_2_O_3_. Ren [28] and Huang et al. [29] proved the hard Al inclusions were modified into CeAlO_3_, Ce_2_O_3_ and Ce_2_O_2_S in sequence with the content of Ce increased from 0 to 0.3%, which improved the deformation coordination between the inclusions and the steel matrix [30]. Additionally, Liu et al. [31] found that the addition of Ce can decrease inclusion size and the area fraction. 

Mehrabian et al. [32] demonstrated that the homogeneity of solute distribution can be obviously enhanced and the segregation of chemical elements can be obviously improved by RE treatment. It was proved that heterogeneous nucleation could increase the nucleation rate and refine the solidification structure with the formation of RE compounds, such as Ce_2_O_3_, Ce_2_O_2_S and La_2_O_3_ [33,34,35]. Waudby et al. [36,37] improved the microstructure and performance of steels by changing the location of non-metallic inclusions and/or nucleation by adding rare earth elements (including Ce, La and Y) into the molten steel; however, industry practices have shown that Ce will block the nozzle during steelmaking [38,39,40,41] and consequently, the Zr element is used to replace the Ce element with a similar effect in oxygen affinity, grain refinement and particle refinement [42]. Shi et al. [43] showed that tested steel with Zr can refine the inclusions and spheroid the second phase in Zr micro-alloyed steel, but they did not investigate the effect of Zr on the steel for SSC resistance. Oxide metallurgy [44] is a process that retains a controllable small content of oxygen in molten steel in the deoxidation process, and as an effective element, it reacts in a metallurgical reaction with the Zr element. 

In this paper, the properties and microstructure of the experimental steels were compared with those produced by the oxide metallurgical process and the traditional aluminum deoxidation process, respectively. The purpose was to try to use the oxide metallurgy process to form oxides or sulfides with a fine size, dispersion distribution, controllable composition, and high melting point, as well as their composite compounds in molten steel, so as to avoid the production of Al_2_O_3_ inclusions in the Al deoxidation process, while attempting to explain the effect of inclusion on strength, toughness and SSC resistance.

## 2. Experimental Method

### 2.1. Preparation of the Experimental Steels

Based on the thermodynamic analysis and calculation of deoxidation for alloy elements, the chemical composition of the experimental steels was smelted in a medium frequency vacuum induction furnace, as presented in Table 1, and the other contents were iron. The steel smelted by the process of Al deoxidation is hereinafter called Al-Steel, and the steel smelted by Zr deoxidation is hereinafter referred to as Zr-Steel. The deoxidation process of the Al-Steel imitated the Al deoxidation smelting technology currently used in industry. The raw materials for smelting were placed into the furnace at one time, an appropriate amount of Al line was added according to the actual situation during the refining process. The Zr-Steel used the oxide metallurgy process, the deoxidized raw materials were placed into the secondary feeding bin of the vacuum induction furnace according to the deoxidization capacity. During the alloying after electromagnetic stirring and argon filling, the deoxidized raw materials were added to the molten steel after temperature control and oxygen control. After the composition was uniform, the steel was poured out.

### 2.2. The Hot Rolling and Heat Treatment of the Experimental Steels

**Figure 1:**
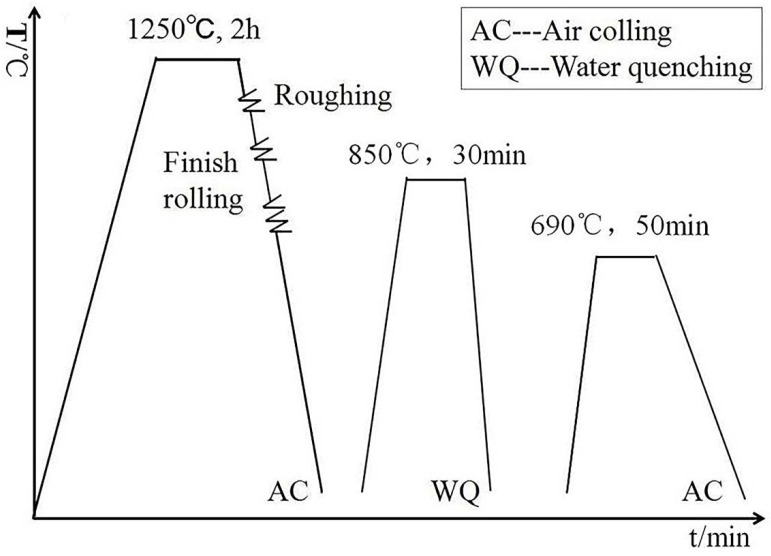
Hot rolling and heat treatment process of experimental steels.

The experimental steels were forged into cube shaped billets with a thickness of 80 mm, then cut, reheated to 1250 °C and held for 2 h, and then billets were rolled to 60 mm through two passes of rough rolling. Finally, the billets were rolled to 14 mm plates for four passes at around 880 °C and then air-cooled to room temperature. Subsequently, the steel plates were reheated to 850 °C and held for 30 min, and then the water was quenched to room temperature. The plates were reheated to 690 °C and held for 50 min, and then they were cooled to room temperature again. The hot-rolled and heat treatment processes are shown in Figure 1. 

### 2.3. Microstructure Observation

When the test steels were finished being quenched according to the above process, the quenched samples of the test steels were taken for an Austenitic structure analysis. The quenched samples were polished to 1500 # with sandpaper, and the grain boundary of the prior austenite structure was corroded with the samples being immersed in a mixed solution of supersaturated picric acid, sodium dodecylbenzene sulfonate and xylene in a constant temperature salt bath furnace at 65 °C. The grain boundary of the prior austenite structure observed by a Leica DM 2500 M optical microscope (OM, Leica Microsystems, Wetzlar, Germany) and the austenite grain size for each process was measured with the Image-J software (Image-J 1.8.0, National Institutes of Health, Bethesda, MD, USA) in 10 fields of view.

After all the processes in 2.2 were completed and before the corrosion test, the test steels were subjected to metallographic structure observation and inclusion detection. In order to observe the metallographic microstructure, the tempered samples were mechanically polished and etched with an alcohol solution containing 4% nitric acid. The morphology of the inclusions was observed by using the field emission electron probe micro-analyzer (EPMA) (JEOL JXA-8530F, Akishima, Japan), and the chemical composition of the inclusions was examined by an energy dispersive X-ray spectrometer (EDS) [45] and surface scanning of the EPMA. The number and size of inclusions were counted by the Image-J software.

### 2.4. Mechanical Properties Experiments

The standard round bar tensile specimens (refer to NACE TM 0177-2005 [46]) had a diameter of 6.35 mm and a gauge length of 25 mm. The tensile tests were carried out on the Instron 100 kN tensile machine with a crosshead speed of 2 mm/min at room temperature (repeated two times). According to the API-5CT, which is the standard for petroleum casing published by the American Petroleum Institute, Charpy impact tests were carried out on an Instron drop weight impact testing machine, using the V-notch specimen (55 mm × 10 mm × 10 mm) at 0 °C (repeated three times). All the tensile and Charpy impact specimens were taken from the steel plates transversely.

### 2.5. NACE-A Test

In order to evaluate the sulfide stress corrosion (SSC) resistance of the test steels, the NACE-A tests were conducted according to the international NACE standard TM 0177-A [46]. The National Association of Corrosion Engineers (NACE) in the United States revised and issued the document as the industry standard for detecting the sulfide stress corrosion cracking resistance of a steel or alloy. The test samples were the standard NACE-A tensile sample with a diameter of 6.35 mm and a gauge length of 25.4 mm, which were immersed in an acidified H_2_S saturated aqueous solution (5.0 wt% NaCl + 0.5 wt% CH_3_COOH dissolved in deionized water). The 85% SMYS (specified minimum yield strength) was the tensile stress of the NACE-A and was loaded at both ends of a constant load, and the fracture time was recorded. The pH value before the experiment was 2.6–2.8 and during the experiment, pure gas N_2_ was used to remove oxygen. The experimental temperature was maintained between 24 ± 3 °C.

## 3. Results and Discussion 

### 3.1. Thermodynamic Analysis of Deoxidation

According to the deoxidation products, the standard Gibbs during deoxidation is shown in Table 2 [47,48], where ΔG^0^ is the standard free energy of oxide formation, J; T is the absolute temperature, K.

**Figure 2:**
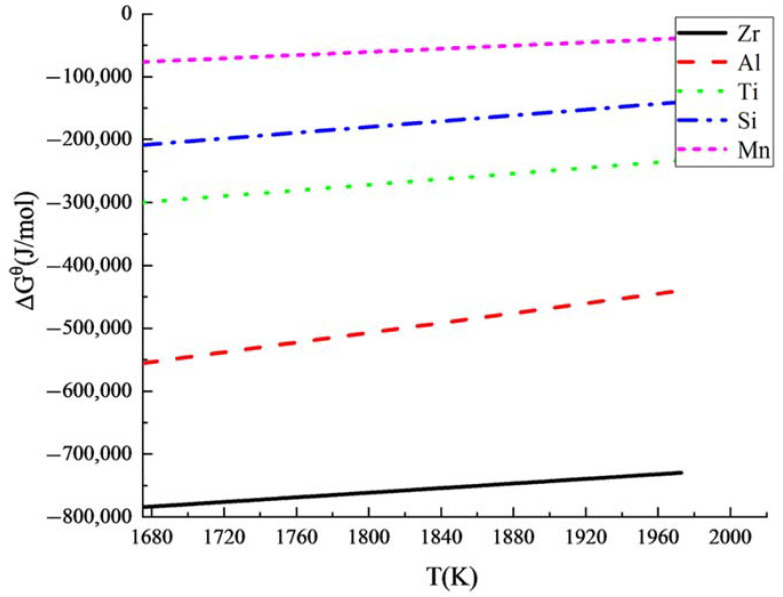
The variation tendency between standard Gibbs free energy and temperature.

The variation tendency of the standard Gibbs with temperature was drawn according to Table 2, and the comparison of the deoxidation ability for the main alloy elements in the experimental steels is shown in Figure 2.

In the metallurgical temperature range, the deoxidation ability is in the order of Ti > Zr > Al > C > Mn. It is inferred from the thermodynamic theory that the deoxidation ability of Zr is higher than that of Al, and the inclusion morphology can be changed with Zr; therefore, it was feasible to use Zr as a deoxidizer instead of Al in the OCTG steel.

**Figure 3:**
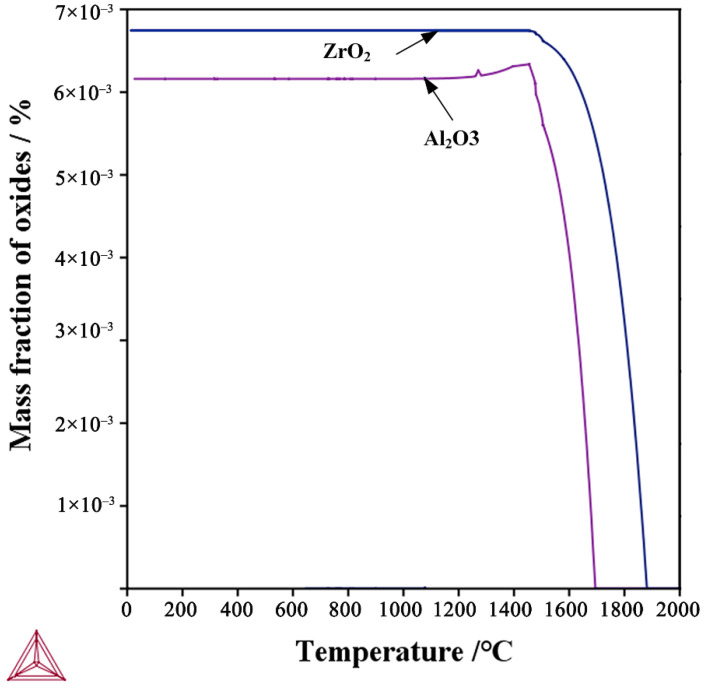
Mass fractions of oxide calculated by ThermalCalc for the Al-Steel and Zr-Steel.

The relevant thermodynamic calculations were performed using the TCFE9 database of Thermo Calc (KTH Royal Institute of Technology, Stockholm, Sweden). The effect of the deoxidation process on the oxide fraction of two experimental steels is shown in Figure 3. The oxide generated in the Al-Steel was mainly Al_2_O_3_, and the oxide generated in the Zr-Steel was mainly ZrO_2_. Additionally, the number of oxides generated in the Zr-Steel was more than that in the Al-Steel. It was shown that the oxide metallurgical process can effectively increase the number of oxide inclusions.

### 3.2. Microstructures

**Figure 4:**
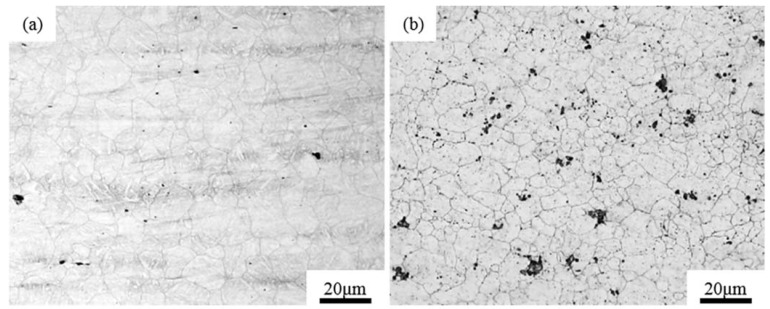
The morphology of prior austenite grain in the same conditions: (**a**) Al-Steel; (**b**) Zr-Steel.

The morphology of the prior austenite grain of the experimental steel was observed after quenching as shown in Figure 4. The average diameter of the prior austenite grain in the Al-Steel was 9.1 μm, relative to Figure 4b, and the grains’ size were slightly uniform. The average diameter of the prior austenite grain of the Zr-Steel was 8 μm. The prior austenite grains of the Zr-Steel were obviously refined, medium with small austenite grains interspersed between large grains. Compared with Figure 4a,b, the number of small austenite grains per unit area in Figure 4b clearly increased. It was demonstrated that the austenite grain size was refined in the specimen treated by the oxide metallurgy process. 

**Figure 5:**
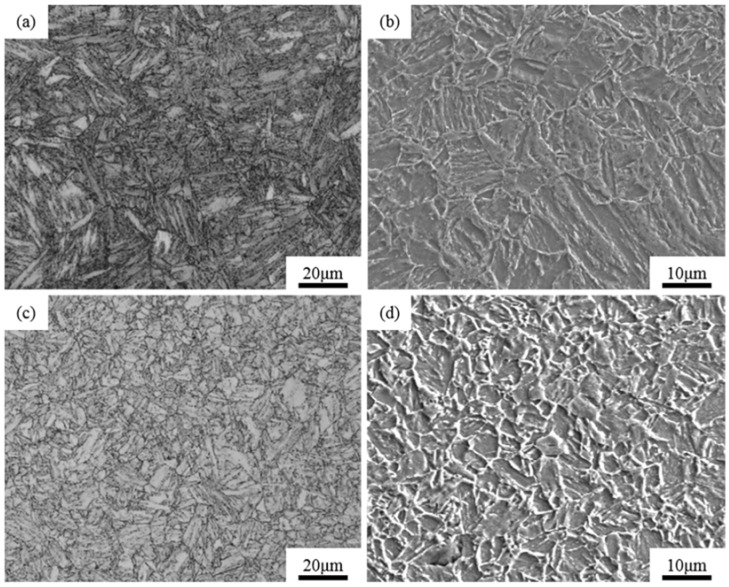
Microstructure images of OM and SEM in the same conditions; (**a**) OM micrographs in Al-Steel; (**b**) SEM micrographs in Al-Steel; (**c**) OM micrographs in Zr-Steel; (**d**) SEM micrographs in Zr-Steel.

The microstructure of the Al-Steel and the Zr-Steel are shown in Figure 5. The microstructure mainly consisted of tempered martensite. The diffusion process of martensite disintegration and carbide transformation occurred during the tempering of the quenched carbon steel, resulting in the formation of a ferrite–cementite structure with a different dispersion and morphology [49]. Because two kinds of experimental steels were tempered at high temperature, the tempered martensite was formed by irregular cementite plate laths, which were filled on the ferrite matrix. The complete martensite structure after high temperature tempering was recognized as the best structure for SSC resistance [50]. The average diameters of the tempered martensite grain in the Al-Steel and the Zr-Steel were 8.2 μm and 3.8 μm, respectively. It was obvious that the microstructure of the Al-Steel was large, but the grain refinement effects of the tempered martensite were quite strong in the Zr-Steel. The martensite grain size was related to the phase transition temperature and prior austenite grain size, whereas the width of the martensite was due to its low transformation temperature, and the length of the martensite lath was restrained by the prior austenite grain boundary [51,52]. The tempered martensite grain of the Zr-Steel was finer, which proves that the oxide metallurgy process can refine the grain, thus promoting the simultaneous improvement of the strength and toughness of Zr-Steel. 

### 3.3. Mechanical Properties

Table 3 shows the mechanical properties of both the experimental steels, where R_σ_ is the yield strength, R is the tensile strength, R_σ_/R is the yield ratio, A is the elongation, A_kv_/0 °C is the impact energy in 0 °C, and NACE- A [SMYS-85%] is the fracture time of NACE-A. The left column of the A_kv_ is the impact energy of each sample, and the right column is their average values. Although the strength of the Al-Steel met the requirements of Q125ksi (yield strength ≥ 862 MPa and a tensile strength ≥ 931 MPa), the toughness was obviously insufficient (impact energy ≥ 100 J), and the fracture time of the NACE-A test was only 28 h, indicating that the sulfide stress corrosion resistance of the Al-Steel was low. The mechanical properties of the Zr-Steel met the requirements of Q125ksi, and it passed the NACE-A test without fracture; thus, it had become a sulfide stress corrosion resistant high-strength steel which met the Q125ksi level.

### 3.4. Inclusions

#### 3.4.1. Inclusions in the Al-Steel

**Figure 6:**
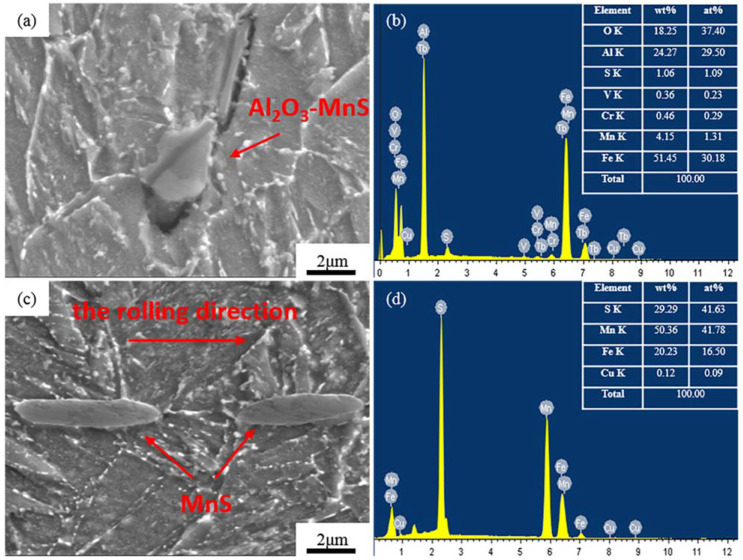
Typical inclusions in Al-Steel. (**a**) The image of Al_2_O_3_-MnS; (**b**) EDS of Al_2_O_3_-MnS; (**c**) the image of MnS; (**d**) EDS of MnS.

The inclusions in the Al-Steel were mostly composed of irregular Al_2_O_3_-MnS (Figure 6a,b) or elongated MnS (Figure 6c,d), respectively. The Al_2_O_3_-MnS angular inclusion in Figure 6a had a long diameter of 3.6 μm, and a short diameter of 2.3 μm. The elongated MnS in Figure 6c (left) had a long diameter of 6.2 μm, a short diameter of 1.1 μm, and an aspect ratio of 5.6. The elongated MnS in Figure 6c (right) had a long diameter of 5.2 μm, a short diameter of 1.1 μm, and an aspect ratio of 4.7. The MnS inclusions were elongated along the rolling direction during the hot rolling. They were easy to form into interconnected banded MnS at the grain boundary. According to the Introduction of this article, the microcrack or the stress would have occurred between the inclusion and matrix because of the difference in the thermal expansion coefficient and mechanical properties between them. Kusche et al. [53] deeply explained the influence of an MnS inclusion on mechanical properties and deformation mechanisms through modeling. The MnS inclusion had two sets of slip surfaces and it was considered that {110} 〈110〉 surface was the main slip system, {100} 〈110〉 was the second slip active surface, and the slip on the {100} plane could be activated. It has been proved by theory and experiments that this activation of multiple slip systems directly leads to the work hardening during the rolling process, and a reduction in the toughness of the steel. This is part of the reason why the impact energy of the Al-Steel was lower than that of the Zr-Steel. The requirements of the SSC resistant OCTG were required to improve the strength, toughness and SSC resistance simultaneously. 

**Figure 7:**
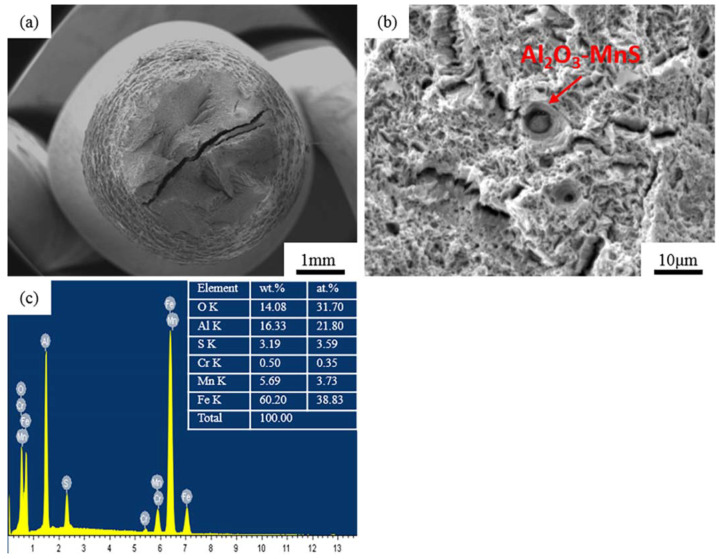
SEM images of the fracture surfaces of Al-Steel after NACE-A. (**a**) Fracture morphology at low magnification; (**b**) inclusion on fracture surface; (**c**) EDS of inclusion of Al-Steel.

The fracture morphology after the Al-Steel NACE-A test is shown in Figure 7. According to Figure 7b, dimple-like structures were formed around the non-metallic inclusions, but different from a dimple, with a lower depth, which is called a “fish eye”, representing quasi-brittle fracture characteristics. There was an obvious crack trend around the inclusion. According to Figure 7c, this inclusion is the typical Al_2_O_3_-MnS inclusion in Al-Steel represented by Figure 6a.

Corresponding to the short fracture time of the Al-steel in the NACE-A test in Table 3, it can be inferred that the typical Al_2_O_3_, MnS and Al_2_O_3_-MnS inclusions would be produced in the Al deoxidation process, and these kinds of inclusions contribute to SSC cracking. Wang et al. [9] found a typical spherical composite inclusion with an Al-O composition, which was the initiation of annular corrosion pits and the steel matrix near the boundary of these inclusions may preferentially dissolve in a corrosive environment. The dissolution of the metal produced anodic ions, which led to the migration of some anions from the electrolyte to the circular boundaries of the inclusions under the concentration gradient, resulting in pits in the horizontal direction, before finally forming circular pits. Shimahashi [54,55] observed pitting corrosion at the position of MnS, which finally formed cavities while some MnS inclusions reacted with a corrosive medium, firstly converted into film form. Further corrosion led to the complete dissolution of the MnS, forming a cavity in the matrix, representing the starting point of stress corrosion. Another form of sulfide stress corrosion cracking caused by MnS is when a part of the MnS cracks under stress and forms microcracks. With the action of stress and environmental corrosion, the cracks then pass through the MnS inclusions. The steel substrate surface under the inclusions is exposed to the medium, and stable corrosion pits begin to form; however, in other cases, the MnS has remained intact. It was reported that the existence of microcracks in the matrix near large inclusions is related to stable pitting corrosion, and that hydrogen atoms are trapped between the inclusion/matrix boundary and aggregated at such defects, reducing the fluidity of H. Because Al deoxidation produces unavoidable inclusions, these kinds of inclusions reduce the strength, toughness and SSC resistance, it is therefore necessary to improve the deoxidation process.

#### 3.4.2. Inclusions of the Zr-Steel

**Figure 8:**
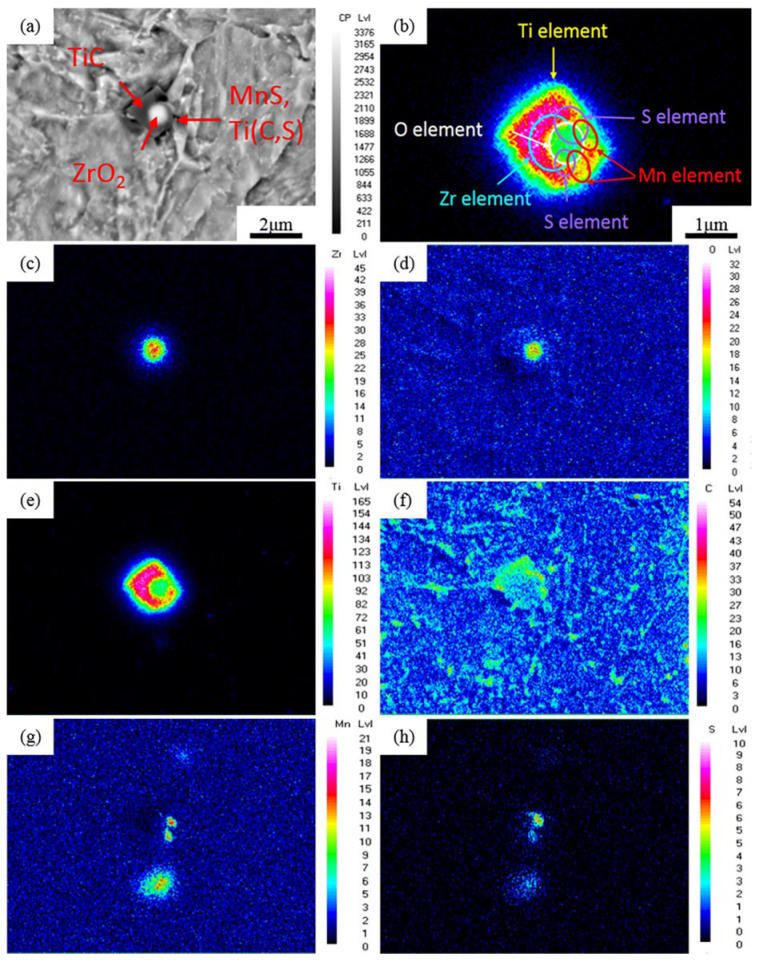
Typical inclusions in Zr-Steel and chemical elements distribution by EPMA. (**a**) Morphology of the inclusion; (**b**) Location of inclusion constituent element distribution; (**c**) Distribution of Zr element; (**d**) O element; (**e**) Ti element; (**f**) C element; (**g**) Mn element; (**h**) S element.

The typical inclusion of the Zr-Steel is shown in Figure 8. Based on Figure 8c,d, it was found that the spherical inclusion in the middle was a ZrO_2_ inclusion with a diameter of 850 nm. The square inclusion on the outside was a compound of Ti, as shown in Figure 8e. Figure 8f presents a square inclusion outside that was a carbon compound of the Ti. The side length of this square inclusion was about 1.6 μm, surrounding the spherical inclusion. With the influence of the Ti on the MnS inclusion, with different temperatures, the addition of Ti can convert MnS into TiS and then into Ti_4_C_2_S_2_ [56], and because the experimental hot rolling temperature was high, this part of the inclusion should have been a MnS and Ti (C, S) inclusion, which was at the edge of the inclusion. It can be extrapolated that the originally elongated MnS inclusion was changed under the process of oxide metallurgy. On account of the crystal lattice of the ZrO_2_, which was produced by oxide metallurgy similar to the MnS (Table 4) [57], the MnS inclusions were easy to be adsorbed near those ZrO_2_ inclusions to form spherical composite inclusions. It can be seen from Figure 8 that the oxide metallurgical process, by adding Zr, can adsorb other inclusions, can effectively refine the inclusion particles, and can have an obvious spheroidization effect on the MnS. Finally, the compound of ZrO_2_-TiC-(Mn, Ti) carbon-sulfur formed in the Zr-Steel.

The number and size of the inclusions in the experimental steels under the same unit area were classified by measuring the long diameter of each inclusion for multiple images through the Image-J software. According to the size grade of the inclusions, the number of inclusions of different sizes per unit area was obtained, as shown in Table 5. The number of inclusions contained in the Al-Steel was less with a total of 22.38; the Zr-Steel contained a total of 68.77 inclusions. The number of inclusions contained in the Zr-Steel was much higher than that in the Al-Steel.

Of the total inclusions in the Zr-Steel, 89.63% were small inclusions (diameter < 3 μm), while the content of small inclusions (diameter < 3 μm) in the Al-Steel was 73.46%. The proportion of the Al-Steel and the Zr-Steel inclusions with a diameter of 2–3 μm was almost the same, but the number clearly increased. The content of the small inclusions with a long diameter of 1–2 μm in the Zr-Steel was 16.2% higher than that of the Al-Steel. The average diameters of the inclusions in the Al-Steel and the Zr-Steel were calculated to be 2.45 μm and 1.65 μm, respectively. It was shown that the Zr-Steel contained more abundant and finer inclusions. 

Using the process of oxide metallurgy with Zr participation in steelmaking, ZrO_2_ inclusions with high melting points form in Zr-Steel. Because of the large affinity between Zr and oxygen in molten steel, the oxide metallurgical chemical reaction between the Zr and a small amount of the O element retained in molten steel can be controlled by a Zr addition, while a part of the generated oxide can remain in the molten steel. Additionally, the density of the generated oxide ZrO_2_ (5.7 g/cm^3^) is higher than that of Al_2_O_3_ (3.9 g/cm^3^) [58,59]. According to Stoke’s law [60], a large number of oxide or sulfide inclusions form with a refined, dispersed, controllable composition and a high melting point in the molten steel, and these composite inclusions will not float or be removed; therefore, the amount and proportion of fine inclusions contained in Zr-Steel smelted by the oxide metallurgy process are greater, which effectively refines the particles. Despite that ZrO_2_ adsorbs other inclusions, due to the amount of ZrO_2_ being substantially increased and with the size itself being quite small, inclusions formed by other alloying elements in steel (e.g., MnS), which can be dispersed and adsorbed to different ZrO_2_ at positions nearby. These are inclusions that should be polymerized together but then be dispersed and adsorbed on different ZrO_2_, resulting in the average size of the inclusions being reduced, reflecting the feature that oxide metallurgy can refine inclusions.

In a proper size range, the increasing number of inclusions is helpful to effectively pin the movement of austenite grain boundaries during the process of liquid steel solidification and subsequent solid-state transformation. As a result, this can prevent the growth of austenite grains. Austenite refinement leads to a tempering martensite refinement, and it plays the role of fine-grain strengthening. 

The composite inclusions formed by the oxide metallurgy process are spheroidized and refined, which makes them relatively “hard”, especially MnS. They are not easy to deform with plastic deformation of the steel matrix under high temperature rolling, and they always maintain a fine spherical or spindle shape before and after deformation. The morphology of inclusions can be controlled by this method, so as to avoid or surmount the anisotropy properties of steel caused by the long-chain distribution of MnS inclusions with the extension and deformation of the steel matrix during the hot rolling processing of conventional steel, so that the longitudinal, transverse and thickness properties of the steel can tend to be consistent [53]. The test results show that compared with the long-chain MnS inclusions formed in the conventional steel smelting process, the tip of the MnS disappears under oxide metallurgy and can improve the low-temperature impact toughness and SSC resistance of steel.

In addition, this kind of phenomenon leads to the void between the inclusions and the matrix being smaller with a large reduction in crack sources. It also leads to the hydrogen atoms not gathering at the same lattice defects, resulting in the hydrogen pressure failing to reach a critical hydrogen pressure value, and SSC not occurring [61]. As the total length of the grain boundary per unit area becomes longer, the energy consumed in the fracture process will also become larger. These aspects promote better SSC resistance in Zr-Steel.

**Figure 9:**
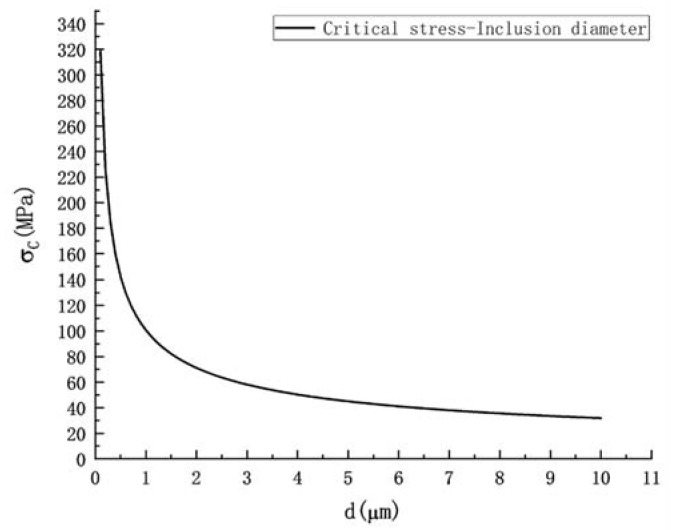
Relationship between inclusion size and critical cracking stress.

The SSC cracking is a kind of HE [1]. A brittle fracture caused by fine inclusions can be explained by considering the classical Griffith theory [62] as follows:

(1) σc=(πEγP(1−ν2)d)12

where, *σ*_c_ is the critical stress, E is the Young modulus, *γ*_P_ is the effective surface energy of the fracture, *ν* is the Poisson ratio, and in this study, d can be roughly regarded as the diameter of the inclusions. According to the test steel, taking [63]: E = 210 GPA, *γ*_P_ = 14 J m^−2^, and *ν* = 0.3. The curve of the critical stress *σ*_c_ and inclusion size d are shown in Figure 9, which shows that the larger the inclusion size is, the smaller the critical cracking stress is, and the easier it is to cause a fracture of the material under the same stress corrosion conditions. According to the average size of the inclusions in the Al-Steel and the Zr-Steel, the critical stresses of the Al-Steel and the Zr-Steel were calculated as follows: *σ*_c-Al_ = 64.35 MPa, and *σ*_c-Zr_ = 78.41 MPa, respectively. This shows that the critical cracking stress of the Al-Steel was smaller and easier to crack. This indicates that the larger the inclusion size is, the easier a brittle fracture will occur, which will reduce the SSC resistance of steel from the perspective of a brittle fracture.

Therefore, the Zr-Steel had excellent toughness, and a high strength and sulfide stress cracking resistance, making the inclusions in the steel a benefit rather than a hindrance. 

## 4. Conclusions

In this paper, the effect of the deoxidation process on the microstructure, inclusion, mechanical properties and sulfide stress corrosion resistance of steel have been investigated. The conclusions are as follows:

(1) The yield strength of the Al-Steel and Zr-Steel was 922 MPa and 939 MPa, respectively; the impact energy was 60 ± 6 J and 132 ± 6 J, respectively; the fracture time of the NACE-A experiment increased from 28 h (Al-Steel) to 720 h (Zr-Steel) without fracture. The oxide metallurgy process improved the sulfide stress corrosion resistance of the steel.
(2) The oxide metallurgy process increased the number of inclusions and decreased the size of the inclusions. These fine particles can pin the austenite grain boundary, refining the prior austenite grain. The average diameter of the austenite grains of the Al-Steel and the Zr-Steel was 9.1 μm and 8 μm, respectively. After the heat treatment, both experimental steels consisted of a tempered martensite structure, and the average grain size of the martensite was 8.2 μm (Al-Steel) and 3.8 μm (Zr-Steel), respectively. The microstructure of the oxide metallurgy process was an ultra-fine grain, contributing to a fine grain strengthening and consumption of the fracture energy. This is the most ideal microstructure for sulfide stress corrosion resistant high-strength steel.
(3) Based on the thermodynamic calculation, it is feasible to deoxidize using Zr instead of Al in steelmaking because of the strong affinity of Zr with oxygen. The number of fine composite inclusions with a high melting point increased due to the oxide metallurgy process by adding zirconium (Zr). The Al-Steel and Zr-Steel contained 22.38 and 68.77 inclusions per unit area, respectively; the fraction of the inclusions with a diameter less than 2μm was 73.46%, and 89.63%, respectively. The average diameter of the inclusions in the Al-Steel (2.45 μm) was larger than that of the Zr-Steel (1.65 μm). An inclusion refinement reduced the lattice defects, and the hydrogen atoms could not be concentrated in the irreversible hydrogen trap, so as to improve the SSC resistance of the steel.
(4) The MnS was obviously spheroidized in the steels treated by the oxide metallurgy process. The stress concentration caused by anisotropy was effectively avoided, and the low-temperature impact toughness and SSC resistance of the steels were improved. The critical stress of fracture increased with the decrease in the inclusion size according to the Griffith theory.

## Figures and Tables

**Table 1:** Chemical composition of experimental steels (wt.%).

No.	C	Si	Mn	P	S	Cr	Ti	V, Mo, Ni, Cu	Al	Zr	O
Al-Steel	0.28	0.29	0.69	0.010	0.009	0.98	0.013	<1.7	0.01	0	0.0029
Zr-Steel	0.27	0.31	0.70	0.011	0.005	0.97	0.011	<1.7	-	0.009	0.0020

**Table 2:** The deoxidation reaction and standard Gibbs free energy.

Deoxidation Reaction	Standard Gibbs Free Energy (J·mol^−1^)	ΔG^θ^ (T = 1873 K) (J·mol^−1^)	Reference
[Zr] + 2[O] = ZrO_2_	ΔG^θ^ = −1,092,000 + 183.7 T	−0.747930 × 10^−6^	[47]
2[Al] + 3[O] = Al_2_O_3_	ΔG^θ^ = −1,205,090 + 387.73 T	−0.478872 × 10^−6^	[48]
[Ti] + 2[O] = TiO_2_	ΔG^θ^ = −675,720 + 224.6 T	−0.255044 × 10^−6^	[47]
[Si] + 2[O] = SiO_2_	ΔG^θ^ = −594,128 + 230 T	−0.163338 × 10^−6^	[48]
[Mn] + [O] = MnO	ΔG^θ^ = −288,773 + 126.82 T	−0.051239 × 10^−6^	[48]

**Table 3:** Mechanical properties of experimental steel.

No.	R_σ_ (MPa)	R (MPa)	R_σ_/R	A (%)	A_kv_/0 °C (J)		NACE-A [SMYS-85%] (h)
Al-Steel	922	964	0.96	15	65		28
					54	60 ± 6	
					61		
Zr-Steel	939	978	0.96	14	129		>720
					135	132 ± 3	
					132		

**Table 4:** Lattice parameters and interplanar distances for ZrO_2_ and MnS (ASTM).

Compound	Crystal Structure	Planes (hkl)	Interplanar Distance (Å)	Crystal Plane Angle, β	Relative Intensity	Lattice Parameters (Å)
ZrO_2_	Monoclinic	002	2.621		20	a = 5.145
		022	1.847	99.2	14	b = 5.207
		113	1.509	3	4	c = 5.311
MnS	Fcc	111	2.612		100	a = 5.224
		220	1.847	90	50	b = 5.224
		222	1.509		20	c = 5.224

**Table 5:** Number of inclusions in different diameters.

No.	1–2 μm	2–3 μm	3–4 μm	4–5 μm	5–6 μm	6–7 μm	7–8 μm	8–9 μm	>9 μm	Total
Al-Steel	10.37	6.07	3.85	1.04	0.30	0.15	0.30	0.15	0.15	22.38
Zr-Steel	43.01	18.63	3.73	1.36	0.68	0.34	0.68	0	0.34	68.77

## Data Availability

The processed data required to reproduce these findings cannot be shared at this time as the data also forms part of an ongoing study.
